# Infectious diseases as a cause of death among cancer patients: a trend analysis and population-based study of outcome in the United States based on the Surveillance, Epidemiology, and End Results database

**DOI:** 10.1186/s13027-021-00413-z

**Published:** 2021-12-31

**Authors:** Muhammed Elhadi, Ala Khaled, Ahmed Msherghi

**Affiliations:** Faculty of Medicine, University of Tripoli, Tripoli, 13275 Libya

**Keywords:** SEER, Infection, Infectious diseases, Cancer, Mortality

## Abstract

**Background:**

Infectious diseases are a major cause of morbidity and mortality among cancer patients. We aimed to determine the incidence of infectious diseases as a cause of death among cancer patients and analyze the trends and risk factors associated with mortality.

**Methods:**

In total, 151,440 cancer patients who died from infectious diseases in the US diagnosed between 1973 and 2014 from the Surveillance, Epidemiology, and End Results program were enrolled. A trend analysis of annual cancer deaths caused by infectious diseases was conducted. Cox proportional hazards model and survival decision tree model were performed.

**Result:**

The most common infectious diseases were pneumonia and influenza (n = 72,133), parasitic and other infectious (n = 47,310) diseases, and septicemia (n = 31,119). The patients’ mean age was 66.33 years; majority of them were male (62%). The overall incidence from 1973 to 2014 showed an insignificant decrease (annual percentage change =  − 0.3, 95% confidence interval [CI] =  − 2.2–1.7, *P* = 0.8). Parasitic and other infectious diseases, including HIV (standardized incidence ratio [SIR] = 1.77, 95% CI = 1.69–1.84), had the highest incidence, followed by septicemia (SIR = 0.84, 95% CI = 0.81–0.88), tuberculosis (SIR = 0.72, 95% CI = 0.51–0.99), and pneumonia (SIR = 0.63, 95% CI = 0.61–0.64). Based on the Cox regression analysis, old black male patients with intrahepatic tumor or acute leukemia of different grades, except the well-differentiated grade, had the highest risk of dying from infectious diseases.

**Conclusion:**

Infectious diseases remain the major cause of morbidity and mortality among cancer patients. Early recognition of risk factors and timely intervention may help mitigate the negative consequences on patients’ quality of life and prognosis, improving the prognosis and preventing early death from infection, which is preventable in most cases.

**Supplementary Information:**

The online version contains supplementary material available at 10.1186/s13027-021-00413-z.

## Introduction

Despite clear evidence that multiple malignancies are prevented, over 18.1 million cancer cases and 9.6 million cancer deaths worldwide are recorded annually [1, 2]. The chances of survival are improving, but cancer continues to be the leading cause of death in the US, with around 608,570 deaths annually [3].

Approximately 10% of new cancer cases worldwide are caused by infections and 85% of them occur in developing countries [4]. Cellular transformation, immune suppression, cell cycle disorders, and increased cell turnover rates are various mechanisms that can increase the risk of cancer caused by viruses [5, 6]. HIV infection triggers the development of malignancies and is marked by severe immunosuppression, which may increase the risk of death due to infectious diseases [7, 8].

Cancer can affect the immune system and other body systems in various ways, thus increasing the risk of infection [9]. These changes in the immune system that regulate the body’s protective mechanisms increase the risk of infection. The increasing risk of infection can be attributed to cancer itself, chemotherapy, unhealthy diet, and other diseases or conditions that are not associated with cancer, such as chronic diseases and aging [10–12]. Therefore, cancer patients are vulnerable to infection.

HIV infection itself is a risk factor for malignancy and a cause of cancer-related death. Patients with a higher risk of cancer-related death are those with chronic immunosuppression (i.e., AIDS); coinfection with oncogenic viruses, bacteria, or parasites; and a high incidence of cancer risk factors associated with lifestyle factors such as smoking, malnutrition, and alcohol [13, 14]. High mortality associated with cancer in people with HIV may be partly attributed to insufficient access to adequate health care or chemotherapy [15].

Pneumonia is a common cause of death in all patient groups. It poses a high risk of morbidity and mortality in immunocompromised cancer patients. A cancer patient’s susceptibility to pneumonia arises from the overall consequences of illness, chemotherapy, and overall immune dysfunction, making them susceptible to pathogen exposure [16]. Pneumonia can cause nearly 10% of hospital admissions among cancer patients, especially those with hematologic malignancies, who have a pneumonia risk exceeding 30% during treatment [17–19]. In reality, pneumonia is the primary cause of death in patients with acute leukemia during the transfusion period [20, 21].

Cytotoxic chemotherapy can produce severe and sometimes long-term neutropenia, which may require hospitalization and can lead to fatal infection [22]. Since neutropenic patients cannot develop vigorous inflammatory reactions, severe infection with mild symptoms and indications can occur, where fever is the primary indicator of infection. This can put neutropenic patients at risk of developing life-threatening infection and sepsis and necessitate the administration of empirical broad-spectrum antimicrobials to reduce the risk of septicemia and, therefore, mortality [23, 24]. The rate of febrile neutropenic-related mortality is up to 11%; in the case of severe sepsis, it can reach 50% [25].

Cancer patients also have a higher risk for tuberculosis (TB). The risk of TB in this patient group is due to immunosuppression caused by chemotherapy and local anatomical alterations in the lungs caused by primary lung cancer or metastasis [26]. Therefore, the prevalence of TB has been increasing in cancer patients, even in those with non-pulmonary cancers [27, 28]. TB-affected cancer patients also have more atypical clinical signs and symptoms than non-cancer patients [29].

No previous studies have investigated infectious diseases as a cause of death in cancer patients among a large population or reported the trends in infectious diseases as a cause of death and the possible risk factors. Therefore, this study aimed to determine the incidence of infectious diseases as a cause of death among cancer patients and analyze the trends and risk factors associated with mortality.

## Methods

### Database

Data used in the analysis were acquired from the Surveillance, Epidemiology, and End Results (SEER) 17 Registry, which includes data on tumors diagnosed between 1973 and 2014 [30]. The SEER*Stat software (version 8.3.5) was used to access the database. The inclusion criteria for the case listing session required that all cases had a known age and all sites were recoded. All cases were defined using the International Classification of Diseases for Oncology (ICD‐O) histology codes. All major types of tumors, including benign and malignant tumors, were included in the study.

Data regarding age, sex, grade, laterality, race, behavior (benign, borderline, in situ, and malignant), marital status, survival time, tumor site, and time of diagnosis were available from the SEER database.

### Outcomes

SEER provides a specific cause of death as mortality codes that are assigned in death certificates. In this instance, patients were considered in the study dataset if their cause of death was infectious diseases, which included the following groups: pneumonia and influenza, septicemia, syphilis, tuberculosis, and other infectious and parasitic diseases, including HIV.

### Statistical analysis

Categorical variables are expressed as percentages, while normally distributed continuous variables are expressed as mean and standard deviation; otherwise, variables are expressed as median and interquartile range.

A trend analysis was conducted to calculate the annual deaths of cancer caused by infectious diseases. First, we extracted the diagnosis year and survival months; then, we calculated the year of death for each patient. The trend analysis was conducted using joinpoint regression analysis with four join points, and the annual percentage change (APC) was determined. Furthermore, the standardized incidence rate was computed using the SEER Stat to identify the incidence of infectious diseases in cancer.

Kaplan–Meier curves were plotted to identify the possible predictors of survival. The log-rank test was used to determine if there was a significant difference between the levels of each variable.

Two models were used in the survival analysis: the Cox proportional hazards model and the survival decision trees model. The 95% confidence interval was calculated using a hazard ratio (HR) ± 1.96 × standard error of the HR. We performed the proportional hazards assumption of Cox regression to ensure that the assumption is satisfied. The accuracy of the model was tested using the concordance index (C-index). The significant variables obtained from the Cox regression analysis were used to construct a nomogram. The nomogram was subjected to 1000 bootstrap resamples for internal validation to correct the C-index and explain the variance. The performance of the nomogram predicting survival was assessed using the C-index, an equivalent variable of the area under the curve (AUC) of the receiver operating characteristic curve for censored data. The nomogram was calibrated for 1, 3, and 5 years by comparing the predicted survival with the observed survival.

The survival decision tree was constructed using rpart; we only used cases with a survival time of more than 0 months. First, we divided our data into training and validation sets. We used a minimum variable at each split of 10 and a maximum depth of 10; then, we pruned the tree to avoid overfitting. The prediction error was calculated using the integrated brier score using the ipred package in R. R Foundation statistical software (R 3.2) was used to perform the data analysis.

## Results

### Patients’ characteristics

Our sample included 151,440 patients who died of infectious diseases (mean age, 66.33 years; 62% male). The most common infectious diseases were pneumonia and influenza (n = 72,133), followed by parasitic diseases and other infectious diseases (n = 47,310), and septicemia (n = 31,119). (Table 1). Majority of the patients were white (n = 123,477) and had malignant tumors (n = 140,081). Approximately 78% of the patients were widowed. The average survival time was 65.31 months; moreover, pneumonia and influenza had the highest survival rate (Table 1). A significant difference was observed in the grade, laterality, race, behavior, and site between the different causes of infections. The most common type of cancer associated with infectious diseases was prostate cancer (n = 20,068), followed by breast cancer (n = 16,676), and Kaposi sarcoma (n = 13,046).

**Table 1:** Baseline characteristics of the included cancer patients who died due to infectious disease

	Overall	Other infectious and parasitic diseases, including HIV	Pneumonia and influenza	Septicemia	Syphilis	Tuberculosis		*P* value
n	151,440	47,310	72,133	31,119	35	843		
Age, mean (SD) in years	66.33 (17.18)	51.51 (17.11)	74.43 (11.82)	70.00 (12.77)	67.77 (17.33)	69.17 (12.92)		< 0.001
Sex (%)								
Female	57,511 (38.0)	10,912 (23.1)	31,595 (43.8)	14,689 (47.2)	15 (42.9)	300 (35.6)		< 0.001
Male	93,929 (62.0)	36,398 (76.9)	40,538 (56.2)	16,430 (52.8)	20 (57.1)	543 (64.4)		
Survival, mean (SD) in months	65.31 (78.17)	38.12 (61.18)	81.09 (81.72)	70.14 (81.85)	74.10 (87.83)	57.43 (69.14)		< 0.001
Grade (%)								
B-cell; pre-B; B-precursor	7818 (5.2)	4899 (10.4)	1898 (2.6)	1001 (3.2)	1 (2.9)	19 (2.3)		< 0.001
Moderately differentiated; Grade II	33,347 (22.0)	5243 (11.1)	19,510 (27.0)	8384 (26.9)	11 (31.4)	199 (23.6)		
NK cell; natural killer cell (1995+)	20 (0.0)	11 (0.0)	4 (0.0)	4 (0.0)	0 (0.0)	1 (0.1)		
Null cell; non-T-non-B	36 (0.0)	22 (0.0)	10 (0.0)	4 (0.0)	0 (0.0)	0 (0.0)		
Poorly differentiated; Grade III	20,022 (13.2)	3458 (7.3)	11,114 (15.4)	5330 (17.1)	3 (8.6)	117 (13.9)		
T-cell	709 (0.5)	428 (0.9)	182 (0.3)	96 (0.3)	0 (0.0)	3 (0.4)		
Undifferentiated; anaplastic; Grade IV	4096 (2.7)	1135 (2.4)	2002 (2.8)	933 (3.0)	2 (5.7)	24 (2.8)		
Unknown	72,008 (47.5)	30,021 (63.5)	29,289 (40.6)	12,279 (39.5)	15 (42.9)	404 (47.9)		
Well differentiated; Grade I	13,384 (8.8)	2093 (4.4)	8124 (11.3)	3088 (9.9)	3 (8.6)	76 (9.0)		
Laterality (%)								
Bilateral, single primary	1198 (0.8)	739 (1.6)	290 (0.4)	160 (0.5)	2 (5.7)	7 (0.8)		< 0.001
Left—origin of primary	20,467 (13.5)	5006 (10.6)	10,792 (15.0)	4542 (14.6)	4 (11.4)	123 (14.6)		
Not a paired site	104,929 (69.3)	35,125 (74.2)	48,181 (66.8)	21,046 (67.6)	23 (65.7)	554 (65.7)		
Only one side—side unspecified	331 (0.2)	114 (0.2)	139 (0.2)	74 (0.2)	0 (0.0)	4 (0.5)		
Paired site, but no information concerning laterality	2177 (1.4)	949 (2.0)	879 (1.2)	326 (1.0)	0 (0.0)	23 (2.7)		
Paired site: midline tumour	31 (0.0)	8 (0.0)	15 (0.0)	8 (0.0)	0 (0.0)	0 (0.0)		
Right—origin of primary	22,307 (14.7)	5369 (11.3)	11,837 (16.4)	4963 (15.9)	6 (17.1)	132 (15.7)		
Race (%)								
American Indian/Alaska Native	738 (0.5)	292 (0.6)	285 (0.4)	148 (0.5)	1 (2.9)	12 (1.4)		< 0.001
Asian or Pacific Islander	7494 (4.9)	2074 (4.4)	3948 (5.5)	1313 (4.2)	0 (0.0)	159 (18.9)		
Black	19,020 (12.6)	8249 (17.4)	5647 (7.8)	4951 (15.9)	10 (28.6)	163 (19.3)		
Unknown	711 (0.5)	213 (0.5)	423 (0.6)	68 (0.2)	0 (0.0)	7 (0.8)		
White	123,477 (81.5)	36,482 (77.1)	61,830 (85.7)	24,639 (79.2)	24 (68.6)	502 (59.5)		
Behavior (%)								
Benign	1067 (0.7)	234 (0.5)	497 (0.7)	331 (1.1)	2 (5.7)	3 (0.4)		< 0.001
Borderline malignancy	2182 (1.4)	525 (1.1)	890 (1.2)	745 (2.4)	0 (0.0)	22 (2.6)		
In situ	8110 (5.4)	2188 (4.6)	4082 (5.7)	1796 (5.8)	4 (11.4)	40 (4.7)		
Malignant	140,081 (92.5)	44,363 (93.8)	66,664 (92.4)	28,247 (90.8)	29 (82.9)	778 (92.3)		
Marital status (%)								
Divorced	10,617 (7.0)	3768 (8.0)	4358 (6.0)	2424 (7.8)	2 (5.7)	65 (7.7)		< 0.001
Married	64,925 (42.9)	12,993 (27.5)	35,789 (49.6)	15,691 (50.4)	17 (48.6)	435 (51.6)		
Separated	2043 (1.3)	566 (1.2)	1036 (1.4)	413 (1.3)	1 (2.9)	27 (3.2)		
Single	34,464 (22.8)	23,609 (49.9)	7133 (9.9)	3607 (11.6)	8 (22.9)	107 (12.7)		
Unknown	9519 (6.3)	2709 (5.7)	4562 (6.3)	2185 (7.0)	0 (0.0)	63 (7.5)		
Unmarried or domestic Partner	39 (0.0)	31 (0.1)	4 (0.0)	4 (0.0)	0 (0.0)	0 (0.0)		
Widowed	29,833 (19.7)	3634 (7.7)	19,251 (26.7)	6795 (21.8)	7 (20.0)	146 (17.3)		

### Trend analysis of patients who died because of infections from 1973 to 2014

**Fig. 1:**
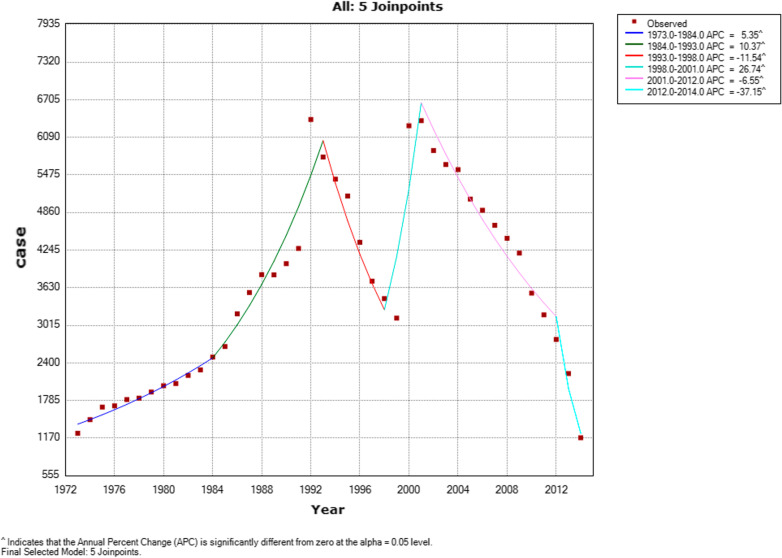
Trend analysis of cancer patients who died because of infectious diseases

Based on the results of the join point analysis, from 1973 to 1984, there was an increase in the number of cases (APC = 5.4%, 95% CI = 3.9–6.8), followed by a significant increase in the rate of deaths from infectious diseases (APC = 10.4%, 95% CI = 8–12.8). However, from 1993 to 1998, a significant drop in the incidence of infectious diseases was observed (APC = 11.5%, 95% CI =  − 17.5 to − 5.7) (Fig. 1). In the next 3 years, a significant increase was observed in the number of deaths caused by cancer that overshot the previous years (APC = 26.74%, 95% CI = 3.7–54.9). From 2001 to 2012 and from 2012 to 2014, a significant decrease was noted in the number of cancer patients who died due to infectious diseases (Table 2). However, the overall trend from 1973 to 2014 showed an insignificant decrease (APC =  − 0.3, 95% CI =  − 2.2–1.7, *P* = 0.8).

**Table 2:** Annual percentage change of deaths caused by infectious diseases

Segment	Lower endpoint	Upper endpoint	APC	Lower CI	Upper CI	Test statistic (t)	Prob >|t|
1	1973	1984	5.4^	3.9	6.8	7.9	0.0
2	1984	1993	10.4^	8.0	12.8	9.3	0.0
3	1993	1998	− 11.5^	− 17.0	− 5.7	− 4.0	0.0
4	1998	2001	26.7^	3.7	54.9	2.4	0.0
5	2001	2012	− 6.5^	− 8.0	− 5.1	− 8.9	0.0
6	2012	2014	− 37.1^	− 48.6	− 23.2	− 4.8	0.0

### Incidence of infectious diseases

**Fig. 2:**
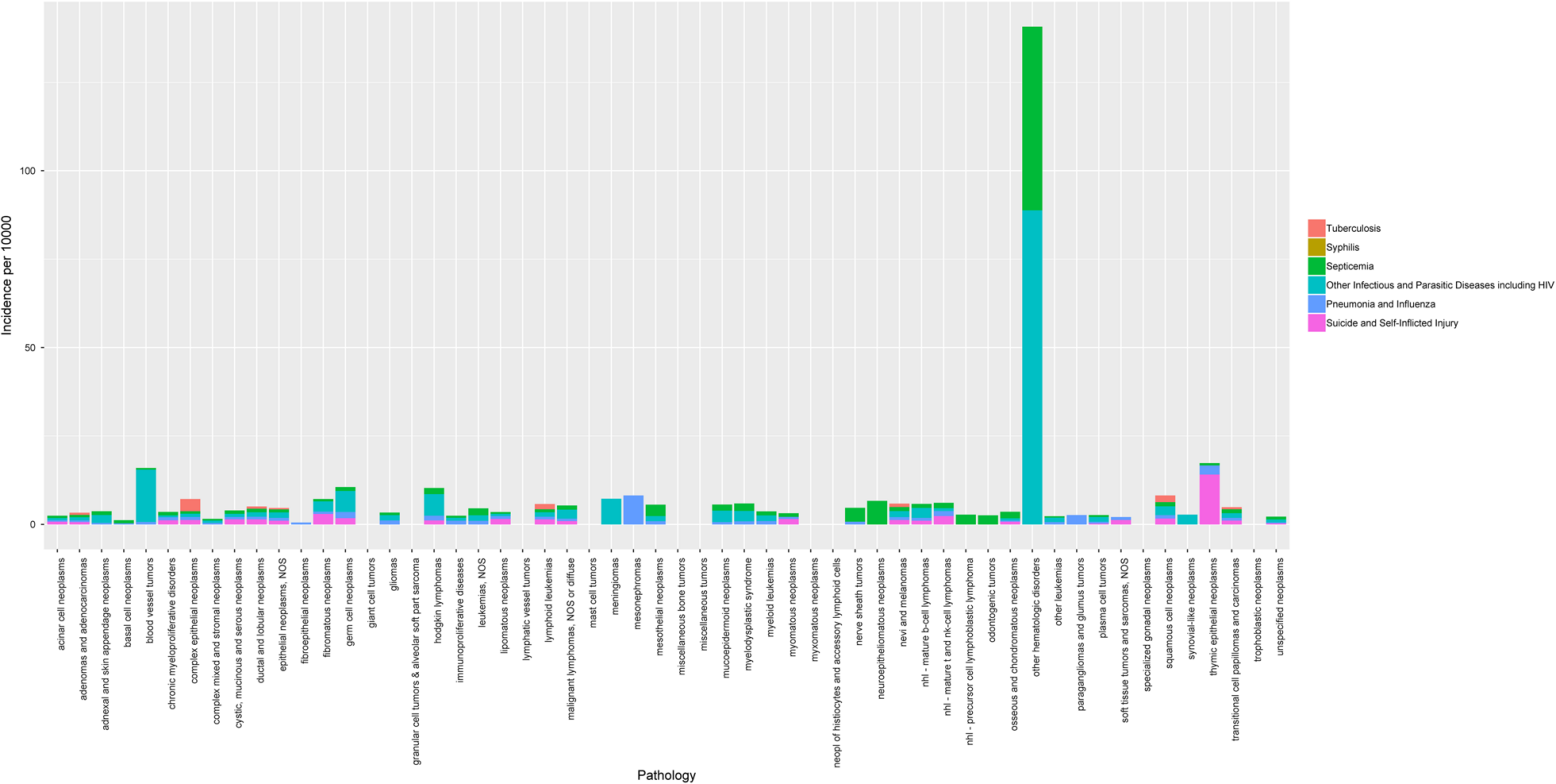
Incidence of reported infectious diseases in cancer patients based on its pathology

The infectious diseases with the highest incidence were parasitic and other infectious diseases, including HIV (standardized incidence ratio [SIR] = 1.77, 95% CI = 1.69–1.84), followed by septicemia (SIR = 0.84, 95% CI = 0.81–0.88), tuberculosis (SIR = 0.72, 95% CI = 0.51–0.99), and pneumonia (SIR = 0.63, 95% CI = 0.61–0.64). Patients with blood vessel tumors including Kaposi Sarcoma had the highest incidence of parasitic disease and HIV infection (SIR = 88.83, 95% CI = 2.25–494.9) (Fig. 2).

The highest SIR of tuberculosis was in complex epithelial neoplasms (SIR = 3.41, 95% CI = 0.09–19.01), followed by squamous cell neoplasms (SIR = 1.86, 95% CI = 0.96–3.25), and lymphoid leukemia (SIR = 1.42, 95% CI = 0.04–7.9) (Fig. 2).

For septicemia, patients with hematologic tumors other than leukemia, lymphoma, plasma cell tumors, and mast cell tumors had the highest incidence of septicemia, estimated to be 51.89% per 100 patients (SIR = 51.9, 95% CI = 1.31–289.16), followed by nerve sheath tumors (SIR = 3.91, 95% CI = 0.1–21.7), and mesothelial neoplasms (SIR = 3.21, 95% CI = 0.39–11.60). Meanwhile, for pneumonia and influenza, the mesonephromas had the highest incidence (SIR = 8.17, 95% CI = 0.21–45.6) (Fig. 2).

### Survival analysis of infectious diseases

**Fig. 3:**
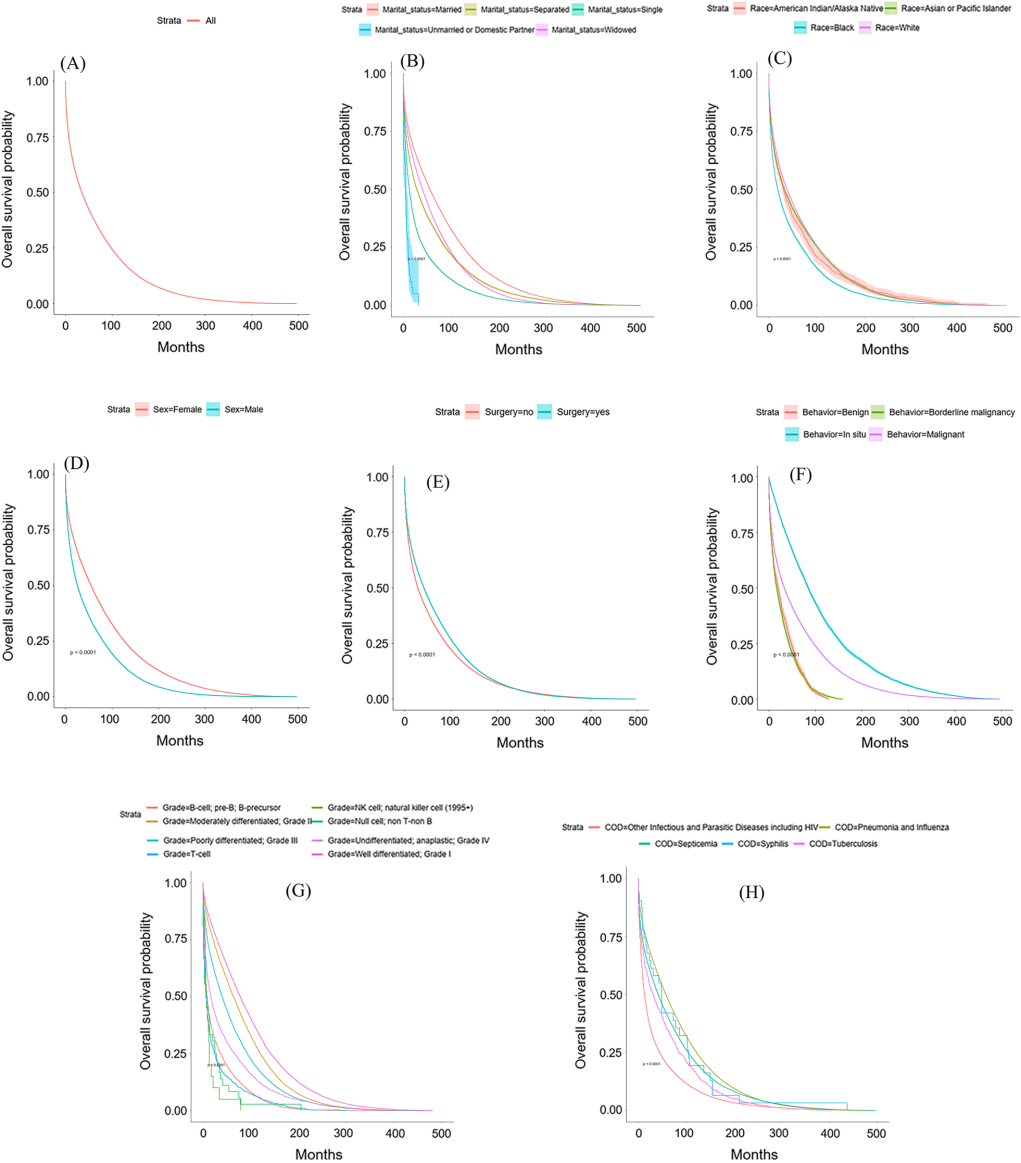
Kaplan Meier survival analysis of infectious diseases

A significant difference was observed in survival between men and women (*P* < 0.0001), different organisms, race, and marital status (*P* < 0.0001). Furthermore, tumor characteristics, including behavior and grade, had significantly different survival according to its level (Fig. 3).

Based on the Cox regression analysis, old black men with intrahepatic tumor or acute leukemia of different grades, except the well-differentiated grade, had the highest risk of dying from infectious diseases (Additional file 3: Table 1). Basal cell neoplasms had the highest significant risk of mortality from infections (HR = 1.33, SE = 0.14, *P* = 0.04) (Additional file 3: Table 1).

### Nomogram for predicting the 1-, 3-, and 5-year survival probability

A nomogram was constructed using significant variables in the Cox regression analysis. The C-index values for the nomogram were 0.85 (95% CI = 0.700–0.9) in the training dataset and 0.87 (95% CI = 0.7–0.9) in the validation set (Additional file 1: Fig. 1). The calibration plots revealed little or no difference between the nomogram prediction and actual observation for the 1-, 3-, and 5-survival years (Additional file 2: Fig. 2).

### Identification of risk groups with prognostic median survival

**Fig. 4:**
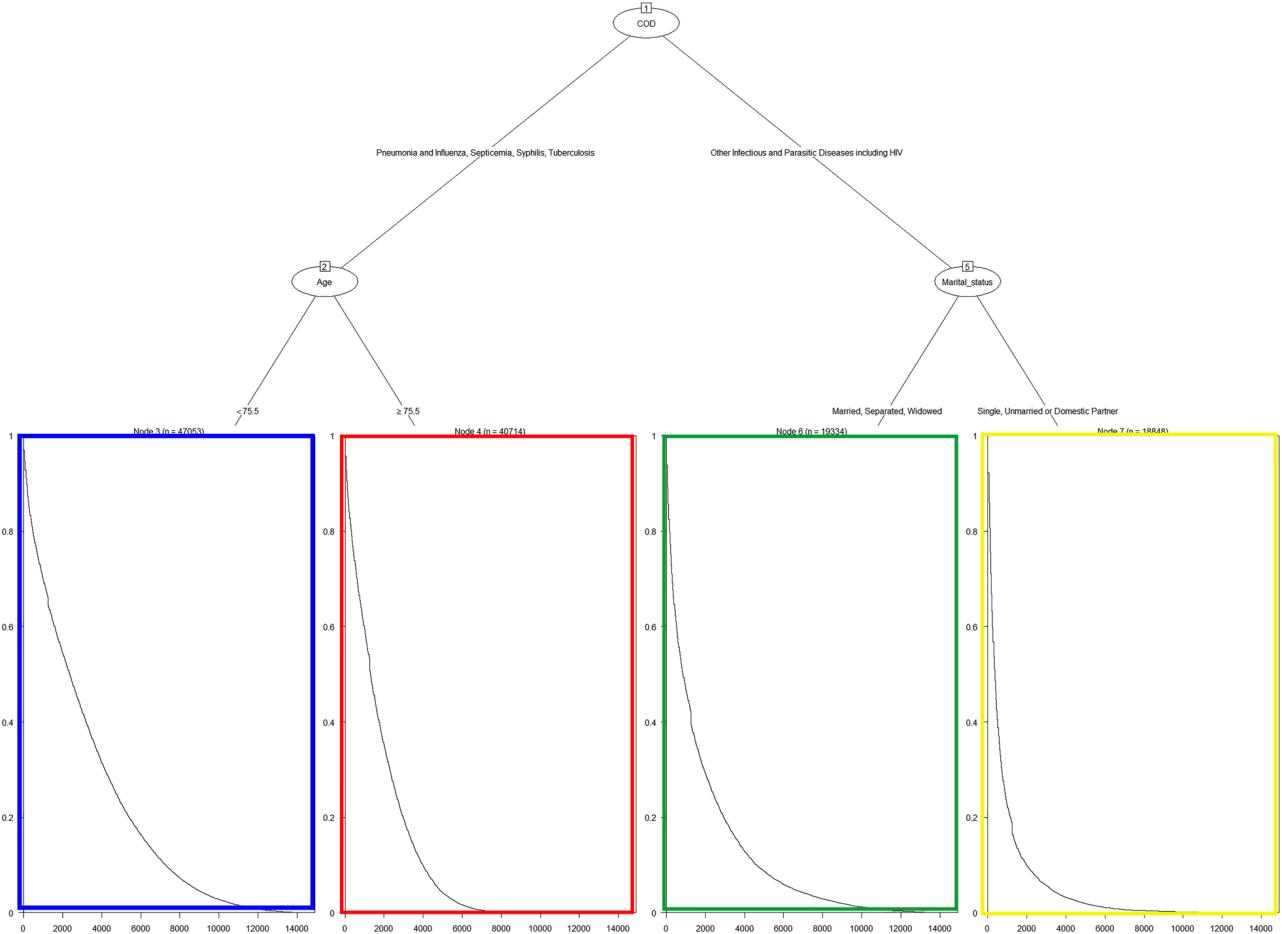
Survival decision tree identifying the four groups with their respective predicted survival. It achieved a brier score of 0.2

The decision tree identified four risk groups for death from infectious diseases. The first group (blue in Fig. 4) included patients with pneumonia and influenza, septicemia, syphilis, and tuberculosis aged < 75.5 years, with a median survival of 2370 days. The second comprised patients aged > 75.5 years with a median survival of 1290 (red in Fig. 4). The third and fourth groups included cancer patients infected with parasitic, HIV, and other infectious diseases and were divided into subgroups based on marital status: married, separated, or widowed (median survival = 840 days) and single, unmarried, or domestic partner (median survival = 360 days) (Fig. 4).

## Discussion

This study determined the incidence of death due to infectious diseases among cancer patients. We also identified the risk factors associated with death due to infectious diseases among 151,440 patients. The most common infectious diseases were pneumonia and influenza. The average survival was 65.31 months; moreover, pneumonia and influenza had the highest survival. The most common cancer associated with infectious diseases was prostate cancer, followed by breast cancer, and Kaposi sarcoma. A significant difference was found in the survival between men and women (*P* < 0.0001), different organisms, race, and marital status (*P* < 0.0001). Old black men with intrahepatic tumor or acute leukemia of different grades, except the well-differentiated grade, had the highest risk of dying from infectious diseases. Basal cell neoplasms had the highest risk of mortality from infections.

To our knowledge, no previous population‐based studies have determined the incidence and risk factors associated with the risk of death due to infectious diseases among cancer patients in the US. Our study found that pneumonia and influenza are the most common infectious diseases causing death among cancer patients, which is a common cause of hospital admission in 10% of cancer patients, especially in those with hematological malignancies. Cancer patients are at greater risk of contracting life-threatening infections, especially pneumonia; some studies reported that 50% of septic shock in cancer patients is caused by bacterial pneumonia [31]. New immunosuppressive therapies produce a variety of immune vulnerabilities that form the basis of opportunistic infections. We also observed a significant decrease in the incidence of infectious diseases in the 1990s and from 2001 to 2014 due to the existence of newer antimicrobials, which also raised concerns about antimicrobial resistance [32]. However, there is a need for vaccinations against viral and bacterial causes of common infections to reduce the burden of antimicrobial resistance [33]. We also found that despite being the major cause of death due to infection, pneumonia and influenza had the highest survival period.

The mean age of the patients was 66.3 years, and most of them were male. The most common type of cancer associated with infectious diseases was prostate cancer. This may be because pneumonia typically affects older patients compared to younger patients, as older patients are usually hospitalized for pneumonia [34]. Another possibility is that androgen deprivation therapy used for prostate cancer increases the risk of pneumonia and hospitalization [35].

Patients with acute leukemia of different grades, except the well-differentiated grade, had a high risk of dying from infectious diseases. Infectious complications are the primary causes of death in patients with acute leukemia [36]. Cancer patients who undergo cytotoxic therapy are at risk for developing infections attributable to the colonizing bacteria or fungi translocating through the mucosal surfaces. In most cases, the earliest and only symptom of infection can be neutropenic fever [25]. Therefore, there is a need to evaluate patients at risk of severe infection. To prevent the escalation of sepsis syndrome and likely death, it is essential to promptly detect neutropenic fever and start empiric systemic antimicrobial therapy [37]. Therefore, risk assessment must be conducted to develop an effective treatment plan.

We found that breast cancer was the second most common type of cancer associated with infectious diseases as a cause of death. Infection is a common cause of breast cancer-related hospitalization, a major cause of morbidity, and an independent predictor of mortality [38]. There are several reasons for the higher infectious disease-related mortality among breast cancer patients. Lymphedema resulting from surgical excision of lymph nodes and/or radiation occurs in 49% of patients [39], which predisposes patients to recurrent infection [40]. Chemotherapy-induced neutropenia is another risk factor for life-threatening infections and sepsis [41]. Several international guidelines, including the American Society of Clinical Oncology (ASCO), recommend granulocyte colony-stimulating factor (G-CSF) and/or antimicrobial agents for breast cancer patients with febrile neutropenia risk to avoid sepsis and severe infection that can result in patient morbidity and mortality [42, 43]. Therefore, early risk assessment and comprehensive evaluation are needed to define febrile patients at risk of severe infection and provide early prophylaxis, which can substantially reduce mortality and morbidity associated with infection and breast cancer patients.

When we evaluated the standardized incidence rate, we found that the incidence of infectious diseases as a cause of death was highest among those with parasitic and other infectious diseases, including HIV, followed by septicemia, tuberculosis, and pneumonia. The development of malignant tumors in HIV patients is one of the most challenging obstacles in the treatment of these patients. Between 30 and 40% of HIV-infected patients are susceptible to developing any cancer type [44]. In fact, Kaposi sarcoma was the third most common type of cancer associated with infectious diseases according to the results of 13,046 patients affected, one of the most common types of cancers in HIV patients [45]. Additionally, patients with vascular tumors that include various tumors, especially Kaposi sarcoma, had the highest incidence of parasitic and HIV infection as a cause of mortality. The trend analysis showed that and period between 1984 and 1993 has a substantial increase in infectious diseases, which is likely related to the HIV epidemic and AIDS defined illness [46, 47]. This was clearly observed in line with HIV infection epidemiology in the USA [48, 49]. Since the mid-1990s, there has been a significant drop in HIV mortality and the prevalence of opportunistic infections in developed countries as a result of widespread antiretroviral therapy (ARV) [50, 51]. This is in line with our results that indicated a decreasing trend in death due to infectious diseases between 1993 and 1998.

African American cancer patients were more likely to die from infection than other races. This ethnic disparity might be attributed to socioeconomic adversity, economic status, ability to access appropriate cancer care, and behavioral and lifestyle differences [52, 53]. Due to insufficient evidence in the SEER database, we were unable to determine the effect of demographic, behavioral, and lifestyle factors on racial disparity in the survival of cancer patients with infectious diseases.

We also found that older men are at a higher risk of death due to infectious diseases among cancer patients. Aging is associated with decreased immune system capacity to adapt appropriately to pathogens and avoid the continued development of tumors, resulting in poor prognosis in these patients [54]. In addition, it puts elderly people at higher risk for opportunistic infections, especially those with hematologic malignancies who are receiving immunosuppressive therapy, which develops neutropenia-related treatment [55]. Additionally, older patients usually have chronic medical conditions that, along with frailty, put them at a higher risk of contracting the infection and developing severe complications, thus increasing the risk of mortality [56].

## Strength and limitations

To our knowledge, this is the first study to investigate the risk of death from infectious diseases among cancer patients in the US. We analyzed the data from a large population-based dataset from 1973 to 2014, a large time period with a wide variety of cases that make the study generalizable to its results. We investigated the risk of death due to infectious diseases among 151,440 cancer patients with different stages and grades. We evaluated the trends for infectious diseases over a long time, which indicated the effect of new antimicrobial and HIV therapy. We also examined the risk factors associated with a higher risk of death due to infectious diseases. However, this study has several limitations. First, the study was conducted over an extensive period of time without identifying the specific baseline, risk factors, or treatment details. Therefore, we cannot determine the appropriate antibiotic prophylaxis and how neutropenic patients’ survival specifically differs from that of others. Second, we did not separately evaluate HIV and instead assessed this condition along with parasitic infection, which may not help distinguish both in terms of survival and related trends in which we draw observations based on the currently available data. The most important limitation of the SEER database is the absence of specific details on chemotherapy types and the loss of long-term follow-up on toxicities and antimicrobial drugs. Details on chemotherapy types and regimens would help distinguish which patients will greatly benefit from reducing immunity or whether cancer itself has an immunosuppressive effect. Finally, we used the cause of death to determine whether it was an infectious disease as SEER does not detail if the patient has had an infectious disease episode and survived from it; it only provided data on patients who died due to infectious diseases. This may limit the interpretability of the results on the morbidity of patients with infectious diseases, as the study focused mainly on mortality associated with infectious diseases.

## Conclusion

Infectious diseases remain the major cause of morbidity and mortality among cancer patients. We found that old black men with intrahepatic tumor or acute leukemia of different grades, except the well-differentiated grade, had the highest risk of dying from infectious diseases. Therefore, early recognition of risk factors and timely intervention may help mitigate the negative consequences on patients’ quality of life and prognosis, which can improve the prognosis and prevent early death from infection, which is preventable in most cases, as shown by the trends that provide insight into the reduction of the infection-related cause of death with the emergence of new antimicrobial and immune-stimulating medications.

## Supplementary Information

**Additional file 1. Supplementary Table 1.** Results of Cox proportional hazard regression analysis to identify the risk for mortality due to infectious diseases.

**Additional file 2. Supplementary Figure 1.** Nomogram Components.

**Additional file 3. Supplementary Figure 2.** Calibration plots of nomogram.

## Data Availability

All data associated with this study is available at www.seer.cancer.gov.
