# Supplementary figures and images for: Infectious diseases as a cause of death among cancer patients: a trend analysis and population-based study of outcome in the United States based on the Surveillance, Epidemiology, and End Results database
Source: Infect Agent Cancer. 2021 Dec 31;16:72. doi: 10.1186/s13027-021-00413-z (PMC8719405; doi:10.1186/s13027-021-00413-z)

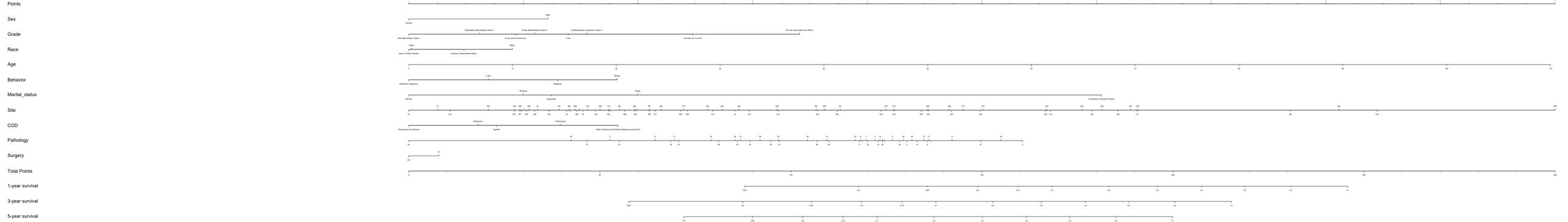

Supplement: Supplementary file 1 — Additional file 1. Supplementary Table 1. Results of Cox proportional hazard regression analysis to identify the risk for mortality due to infectious diseases. [file 13027_2021_413_MOESM1_ESM.pdf]
