# Supplementary material for: Infectious diseases as a cause of death among cancer patients: a trend analysis and population-based study of outcome in the United States based on the Surveillance, Epidemiology, and End Results database
Source: Infect Agent Cancer. 2021 Dec 31;16:72. doi: 10.1186/s13027-021-00413-z (PMC8719405; doi:10.1186/s13027-021-00413-z)
Supplement: Supplementary file 3 — Additional file 3. Supplementary Figure 2. Calibration plots of nomogram. [file 13027_2021_413_MOESM3_ESM.docx]

**Supplementary Table 1. Results of Cox proportional hazard regression analysis to identify the risk for mortality due to infectious diseases**

|  | Hazard ratio | standard error | *p-value* |
| --- | --- | --- | --- |
| Female | 1.00 |  |  |
| Male | 1.34 | 0.01 | < 2e-16 |
| B-cell; pre-B; B-precursor | 1.00 |  |  |
| Moderately differentiated; II | 0.94 | 0.02 | 0.00 |
| NK cell; natural killer cell (1995+) | 1.59 | 0.23 | 0.04 |
| Null cell; non-T-non B | 1.46 | 0.17 | 0.02 |
| Poorly differentiated; III | 1.05 | 0.02 | 0.00 |
| T-cell | 1.11 | 0.04 | 0.01 |
| Undifferentiated; anaplastic; IV | 1.17 | 0.02 | 0.00 |
| Well differentiated; I | 0.80 | 0.02 | < 2e-16 |
| American Indian/Alaska Native | 1.00 |  |  |
| Asian or Pacific Islander | 0.88 | 0.04 | 0.00 |
| Black | 1.09 | 0.04 | 0.02 |
| White | 0.89 | 0.04 | 0.00 |
| Age | 1.02 | 0.00 | < 2e-16 |
| Benign | 1.00 |  |  |
| Borderline malignancy | 0.65 | 0.07 | 0.00 |
| In situ | 0.78 | 0.06 | 0.00 |
| Malignant | 0.90 | 0.06 | 0.08 |
| Married |  |  |  |
| Separated | 1.34 | 0.01 | < 2e-16 |
| Single | 1.61 | 0.01 | < 2e-16 |
| Unmarried or Domestic Partner | 3.15 | 0.16 | 0.00 |
| Widowed | 1.27 | 0.01 | < 2e-16 |
| Acute Lymphocytic Leukemia | 1.00 |  |  |
| Acute Monocytic Leukemia | 1.69 | 0.14 | 0.00 |
| Acute Myeloid Leukemia | 1.08 | 0.07 | 0.25 |
| Aleukemic, Subleukemic and NOS | 0.74 | 0.09 | 0.00 |
| Anus, Anal Canal and Anorectum | 0.42 | 0.06 | < 2e-16 |
| Appendix | 0.45 | 0.12 | 0.00 |
| Ascending Colon | 0.33 | 0.06 | < 2e-16 |
| Bones and Joints | 0.43 | 0.10 | 0.00 |
| Brain | 0.98 | 0.07 | 0.75 |
| Breast | 0.27 | 0.06 | < 2e-16 |
| Cecum | 0.32 | 0.06 | < 2e-16 |
| Cervix Uteri | 0.25 | 0.06 | < 2e-16 |
| Chronic Lymphocytic Leukemia | 0.39 | 0.06 | < 2e-16 |
| Chronic Myeloid Leukemia | 0.71 | 0.07 | 0.00 |
| Corpus Uteri | 0.23 | 0.06 | < 2e-16 |
| Cranial Nerves Other Nervous System | 0.55 | 0.08 | 0.00 |
| Descending Colon | 0.30 | 0.07 | < 2e-16 |
| Esophagus | 0.76 | 0.07 | 0.00 |
| Eye and Orbit | 0.29 | 0.09 | < 2e-16 |
| Floor of Mouth | 0.39 | 0.08 | < 2e-16 |
| Gallbladder | 0.56 | 0.09 | 0.00 |
| Gum and Other Mouth | 0.38 | 0.07 | < 2e-16 |
| Hepatic Flexure | 0.33 | 0.07 | < 2e-16 |
| Hodgkin - Extranodal | 0.65 | 0.17 | 0.01 |
| Hodgkin - Nodal | 0.50 | 0.06 | < 2e-16 |
| Hypopharynx | 0.56 | 0.08 | 0.00 |
| Intrahepatic Bile Duct | 1.38 | 0.11 | 0.00 |
| Kaposi Sarcoma | 0.82 | 0.06 | 0.00 |
| Kidney and Renal Pelvis | 0.35 | 0.06 | < 2e-16 |
| Large Intestine, NOS | 0.44 | 0.07 | < 2e-16 |
| Larynx | 0.33 | 0.06 | < 2e-16 |
| Lip | 0.28 | 0.07 | < 2e-16 |
| Liver | 0.96 | 0.06 | 0.53 |
| Lung and Bronchus | 0.68 | 0.06 | 0.00 |
| Melanoma of the Skin | 0.29 | 0.06 | < 2e-16 |
| Mesothelioma | 0.91 | 0.11 | 0.40 |
| Miscellaneous | 0.87 | 0.06 | 0.03 |
| Myeloma | 0.62 | 0.06 | 0.00 |
| Nasopharynx | 0.36 | 0.09 | < 2e-16 |
| NHL - Extranodal | 0.75 | 0.06 | 0.00 |
| NHL - Nodal | 0.63 | 0.06 | 0.00 |
| Nose, Nasal Cavity and Middle Ear | 0.37 | 0.09 | < 2e-16 |
| Oropharynx | 0.54 | 0.11 | 0.00 |
| Other Acute Leukemia | 2.13 | 0.11 | 0.00 |
| Other Biliary | 0.71 | 0.08 | 0.00 |
| Other Digestive Organs | 1.41 | 0.12 | 0.00 |
| Other Endocrine including Thymus | 0.55 | 0.08 | 0.00 |
| Other Female Genital Organs | 0.37 | 0.14 | 0.00 |
| Other Lymphocytic Leukemia | 0.48 | 0.10 | 0.00 |
| Other Male Genital Organs | 0.31 | 0.13 | < 2e-16 |
| Other Myeloid/Monocytic Leukemia | 0.93 | 0.13 | 0.58 |
| Other Non-Epithelial Skin | 0.34 | 0.07 | < 2e-16 |
| Other Oral Cavity and Pharynx | 0.64 | 0.12 | 0.00 |
| Other Urinary Organs | 0.35 | 0.10 | < 2e-16 |
| Ovary | 0.37 | 0.07 | < 2e-16 |
| Pancreas | 1.04 | 0.07 | 0.54 |
| Penis | 0.30 | 0.08 | < 2e-16 |
| Peritoneum, Omentum and Mesentery | 0.62 | 0.16 | 0.00 |
| Pleura | 1.00 | 0.28 | 0.99 |
| Prostate | 0.25 | 0.06 | < 2e-16 |
| Rectosigmoid Junction | 0.29 | 0.06 | < 2e-16 |
| Rectum | 0.31 | 0.06 | < 2e-16 |
| Retroperitoneum | 0.50 | 0.13 | 0.00 |
| Salivary Gland | 0.32 | 0.08 | < 2e-16 |
| Sigmoid Colon | 0.28 | 0.06 | < 2e-16 |
| Small Intestine | 0.43 | 0.07 | < 2e-16 |
| Soft Tissue including Heart | 0.35 | 0.07 | < 2e-16 |
| Splenic Flexure | 0.32 | 0.07 | < 2e-16 |
| Stomach | 0.46 | 0.06 | < 2e-16 |
| Testis | 0.31 | 0.08 | < 2e-16 |
| Thyroid | 0.29 | 0.07 | < 2e-16 |
| Tongue | 0.41 | 0.07 | < 2e-16 |
| Tonsil | 0.41 | 0.07 | < 2e-16 |
| Trachea, Mediastinum and Other Respiratory Organs | 0.69 | 0.15 | 0.01 |
| Transverse Colon | 0.34 | 0.07 | < 2e-16 |
| Ureter | 0.32 | 0.08 | < 2e-16 |
| Urinary Bladder | 0.29 | 0.06 | < 2e-16 |
| Uterus, NOS | 0.37 | 0.16 | 0.00 |
| Vagina | 0.33 | 0.09 | < 2e-16 |
| Vulva | 0.38 | 0.07 | < 2e-16 |
| Acinar cell neoplasms | 1.00 |  |  |
| Adenomas and adenocarcinomas | 0.98 | 0.06 | 0.81 |
| Adnexal and skin appendage neoplasms | 1.05 | 0.10 | 0.58 |
| Basal cell neoplasms | 1.33 | 0.14 | 0.04 |
| Blood vessel tumors | 0.56 | 0.06 | < 2e-16 |
| Chronic myeloproliferative disorders | 1.10 | 0.08 | 0.22 |
| Complex epithelial neoplasms | 0.97 | 0.08 | 0.65 |
| Complex mixed and stromal neoplasms | 0.96 | 0.08 | 0.59 |
| Cystic, mucinous and serous neoplasms | 1.02 | 0.06 | 0.78 |
| Ductal and lobular neoplasms | 0.99 | 0.06 | 0.87 |
| Epithelial neoplasms, NOS | 0.95 | 0.06 | 0.44 |
| Fibroepithelial neoplasms | 1.12 | 0.23 | 0.62 |
| Fibromatous neoplasms | 1.06 | 0.08 | 0.46 |
| Germ cell neoplasms | 0.58 | 0.09 | 0.00 |
| Gliomas | 0.74 | 0.07 | 0.00 |
| Hodgkin lymphomas | 0.70 | 0.07 | 0.00 |
| Immunoproliferative diseases | 0.99 | 0.11 | 0.90 |
| Leukemias, NOS | 0.99 | 0.09 | 0.92 |
| Lipomatous neoplasms | 0.89 | 0.11 | 0.27 |
| Lymphoid leukemias | 0.94 | 0.07 | 0.35 |
| Malignant lymphomas, NOS or diffuse | 0.65 | 0.06 | 0.00 |
| Meningiomas | 1.10 | 0.07 | 0.18 |
| Mesothelial neoplasms | 0.81 | 0.11 | 0.06 |
| Miscellaneous bone tumors | 0.36 | 0.27 | 0.00 |
| Miscellaneous tumors | 0.77 | 0.17 | 0.13 |
| Mucoepidermoid neoplasms | 1.04 | 0.11 | 0.75 |
| Myelodysplastic syndrome | 1.09 | 0.07 | 0.20 |
| Myeloid leukemias | 0.74 | 0.07 | 0.00 |
| Myomatous neoplasms | 0.80 | 0.09 | 0.01 |
| Myxomatous neoplasms | 0.66 | 0.71 | 0.57 |
| Neopl of histiocytes and accessory lymphoid cells | 0.59 | 0.27 | 0.05 |
| Nerve sheath tumors | 0.98 | 0.12 | 0.87 |
| Neuroepitheliomatous neoplasms | 0.63 | 0.17 | 0.01 |
| Nevi and melanomas | 1.05 | 0.06 | 0.48 |
| NHL - mature b-cell lymphomas | 0.74 | 0.06 | 0.00 |
| NHL - mature t and nk-cell lymphomas | 0.76 | 0.07 | 0.00 |
| NHL - precursor cell lymphoblastic lymphoma | 0.55 | 0.15 | 0.00 |
| Osseous and chondromatous neoplasms | 0.73 | 0.12 | 0.01 |
| Other hematologic disorders | 0.83 | 0.15 | 0.21 |
| Other leukemias | 1.00 | 0.09 | 0.98 |
| Plasma cell tumors | 0.96 | 0.07 | 0.54 |
| Soft tissue tumors and sarcomas, NOS | 0.90 | 0.10 | 0.31 |
| Specialized gonadal neoplasms | 1.30 | 0.27 | 0.33 |
| Squamous cell neoplasms | 0.85 | 0.06 | 0.01 |
| Synovial-like neoplasms | 1.25 | 0.26 | 0.38 |
| Thymic epithelial neoplasms | 0.89 | 0.13 | 0.33 |
| Transitional cell papillomas and carcinomas | 1.06 | 0.06 | 0.33 |
| Trophoblastic neoplasms | 0.55 | 0.27 | 0.03 |
| Unspecified neoplasms | 1.10 | 0.06 | 0.16 |
| No surgery | 1.00 |  |  |
| Surgery | 0.94 | 0.01 | < 2e-16 |
